# Driving fatalities on US presidential election days: a reanalysis

**DOI:** 10.1186/s13104-016-2148-6

**Published:** 2016-07-13

**Authors:** Fan Zhang, Peter M. Aronow

**Affiliations:** Department of Biostatistics, Yale University, 60 College Street, New Haven, CT 06520-8034 USA; Department of Political Science and Biostatistics, Yale University, 87 Trumbull Street, New Haven, CT 06520-8301 USA

**Keywords:** Replication, Non-parametric statistics, Public health, Elections, Voting, Driving fatalities

## Abstract

**Background:**

Redelmeier and Tibshirani reported a statistical analysis in the *Journal of the American Medical Association* in 2008 indicating that presidential election days are strongly associated (*P* < 0.001) with an increased risk of driving fatalities (as measured by the number of persons involved in fatal crashes).

**Findings:**

We present evidence indicating that the risk of driving fatalities on presidential election days is neither statistically nor substantively unusual. Although we find weakly suggestive evidence that presidential elections may increase the risk of driving fatalities during election hours, any increase appears to be entirely offset by a lowered risk during non-election hours.

**Conclusions:**

We find weaker support for an association between election days and driving fatalities than was previously reported. Our results suggest caution in evaluating policy prescriptions that presuppose that election days pose an unusual risk to the public.

## Findings

Redelmeier and Tibshirani [1], henceforth RT, reported a statistical analysis indicating that presidential election days are strongly associated (*P* < 0.001) with an increased risk of driving fatalities. Given this positive association between election days and driving fatalities, RT concluded that the results suggest that policy interventions are warranted to alleviate the increased risk posed by election days. We sought to replicate and extend these results to reassess both the robustness of RT’s results and the implications for policy making.

## Methods

RT used data collected from the Fatality Analysis Reporting System over the years 1975–2006. The primary analysis computed a relative risk by taking the number of persons in fatal crashes during election hours on presidential election days from 1976 through 2004, and comparing this to the average of the number of individuals in fatal crashes during the same hours one week before and one week after. We henceforth refer to RT’s test statistic—the risk relative to the same time period in the previous and following week—as the RR (relative risk).^1^ Throughout, in the interest of replication, we will use RT’s test statistic as our basis for inference.

Our replication extended RT’s methodology with an updated dataset from the Fatality Analysis Reporting System; we examine alternative time periods and use an alternative method to characterize uncertainty. Following a finding reported in a later article by Redelmeier and Tibshirani [2], we estimate RRs during non-election hours as well as during the full 24 h on presidential election days.

To assess statistical significance, we estimated RRs for the 100 Tuesdays before and after presidential election days. (We restrict ourselves to Tuesdays as all United States presidential elections are held on Tuesdays, thus conditioning on any day-of-week effects.) Using these 200 RRs, we constructed an empirical null distribution, with non-parametric two-tailed *P* values computed as the proportion of RRs more extreme (i.e., RR greater or 1/RR smaller) than that of presidential election days. This procedure tests against the null hypothesis that election days have an RR consistent with being drawn randomly from the distribution of Tuesdays [3]. We further calculated 95 % Wald-type confidence intervals (CIs) under a normal approximation using the standard deviation of the empirical null distribution as an estimate of the standard error. Throughout, we consider only presidential elections from 1980 through 2008, as data were not available before 1975 or after 2012, precluding estimation for all Tuesdays surrounding the 1976 or 2012 elections.

Our method for characterizing uncertainty differs from RT’s method, which computes *P* values using a binomial test. The binomial test assumes that, under the null hypothesis, driving fatalities occur with equal probability on election days as on the Tuesdays in the week before and the week after. Since the probability of driving fatalities may differ from week to week for reasons that are unrelated to election days, a binomial test may overstate certainty about the risk posed by election days. To evaluate the properties of RT’s binomial test, we calculated the rate at which it rejected the null hypothesis of no effect across the null distribution of non-election day Tuesdays.

## Results

We estimate an RR for election hours during presidential elections from 1980 to 2008 of 1.17 (non-parametric *P* = 0.070, Wald-type 95 % CI [0.99,1.35]). While our point estimate closely matches RT’s, our measures of uncertainty are much larger, suggesting that the binomial test may be anticonservative in this setting. In fact, when applied to the distribution of Tuesdays, we find that RT’s procedure would reject the null hypothesis of no effect of a given day during election hours 43 % of the time, suggesting that it is sensitive to natural week-to-week variation in driving fatalities.

**Fig. 1:**
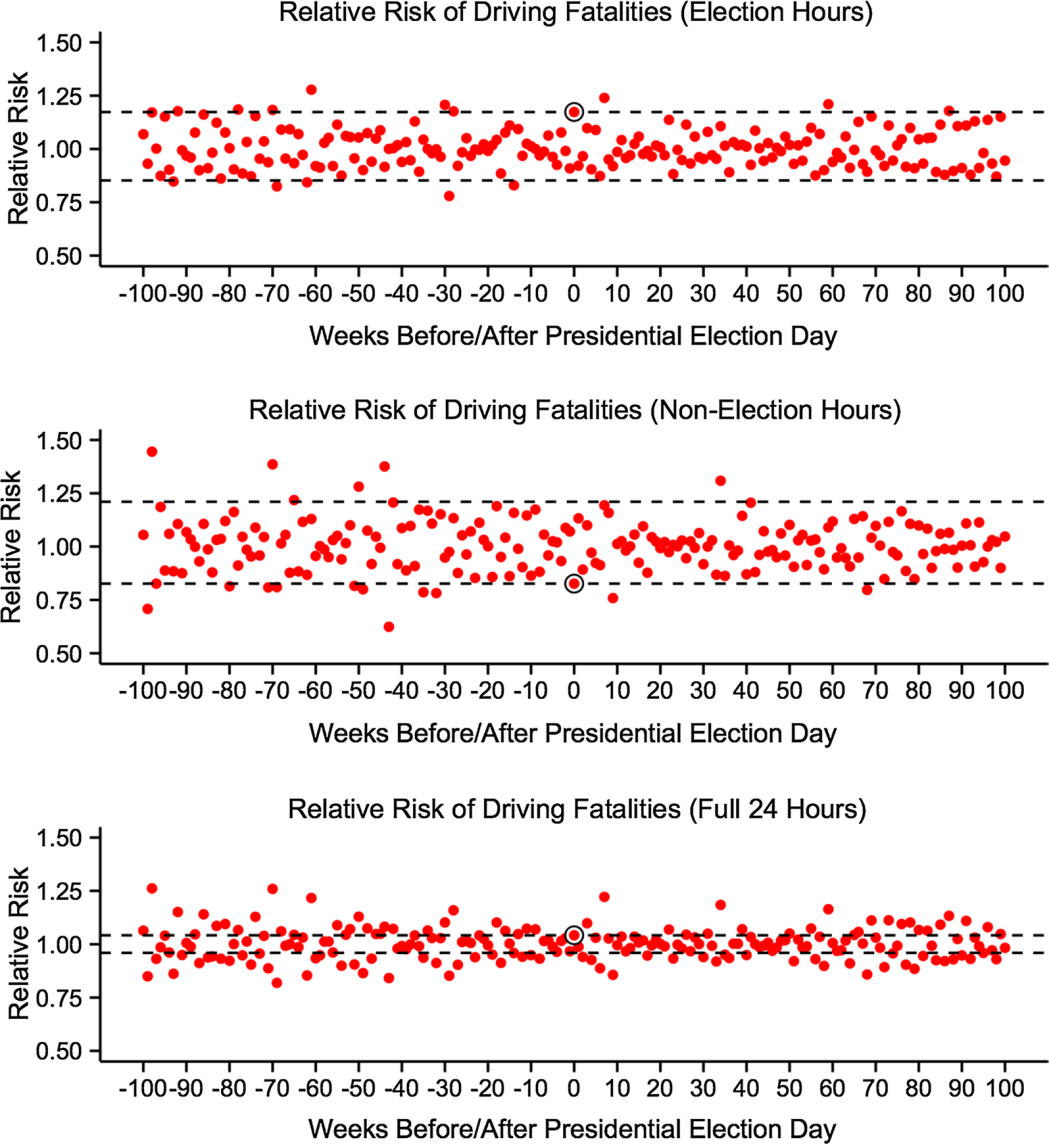
Relative risk (RR) estimates for presidential election days and the 100 preceding and following Tuesdays. *Red dots* indicate RRs for all Tuesdays. *Black circles* indicate RRs and *black dashed lines* indicate RR and 1/RR for presidential election days. The proportion of *red dots* outside *black dashed lines* is equivalent to the reported non-parametric *P* value

Furthermore, we estimate an equally strong negative effect of presidential elections on driving fatalities during non-election hours on election days, with an RR = 0.83 (non-parametric *P* = 0.085, Wald-type 95 % CI [0.60,1.06]). When we included the full 24 h on election days, we found no evidence of an effect of presidential elections, with an RR = 1.04 (non-parametric *P* = 0.570, Wald-type 95 % CI [0.89,1.20]). Figure 1 shows the RRs for election days and the surrounding 200 Tuesdays.^2^

## Discussion

Although we find a suggestive and marginally statistically significant effect (*P* < 0.10) of presidential elections on driving fatalities during election hours, this effect was offset by a lowered risk during non-election hours. Our study shares the same empirical limitations as RT and we would recommend caution in drawing any causal inferences from a non-randomized study. However, as a policy matter, the statistical evidence suggests presidential elections do not appear to be unusual in terms of their associated risk of driving fatalities on election days.

## Abbreviations

CI: confidence interval
RR: relative risk
RT: Redelmeier and Tibshirani

## Footnotes

1 We set aside the question of whether or not the number of persons involved in fatal crashes constitutes an appropriate measure for driving fatalities, and adhere to RT’s usage in the interest of replication. We note that we replicated all of our results using (i) the number of fatal crashes and (ii) the number of fatalities as measured by FARS. Our substantive findings are strengthened in these cases, as we find even weaker statistical associations between election days and fatalities using these alternative metrics.

2 We also computed *P* values for alternative time periods for the null distribution, namely for the surrounding 12, 25 and 50 Tuesdays. For the 12 surrounding Tuesday specifications, we find for election hours, non-election hours, and the full day, respective *P* values of 0.04, 0.04, and 0.46. For 25 Tuesdays, we find respectively 0.06, 0.02, and 0.42. For 50 Tuesdays, we find respectively 0.05, 0.08, and 0.47.
